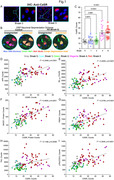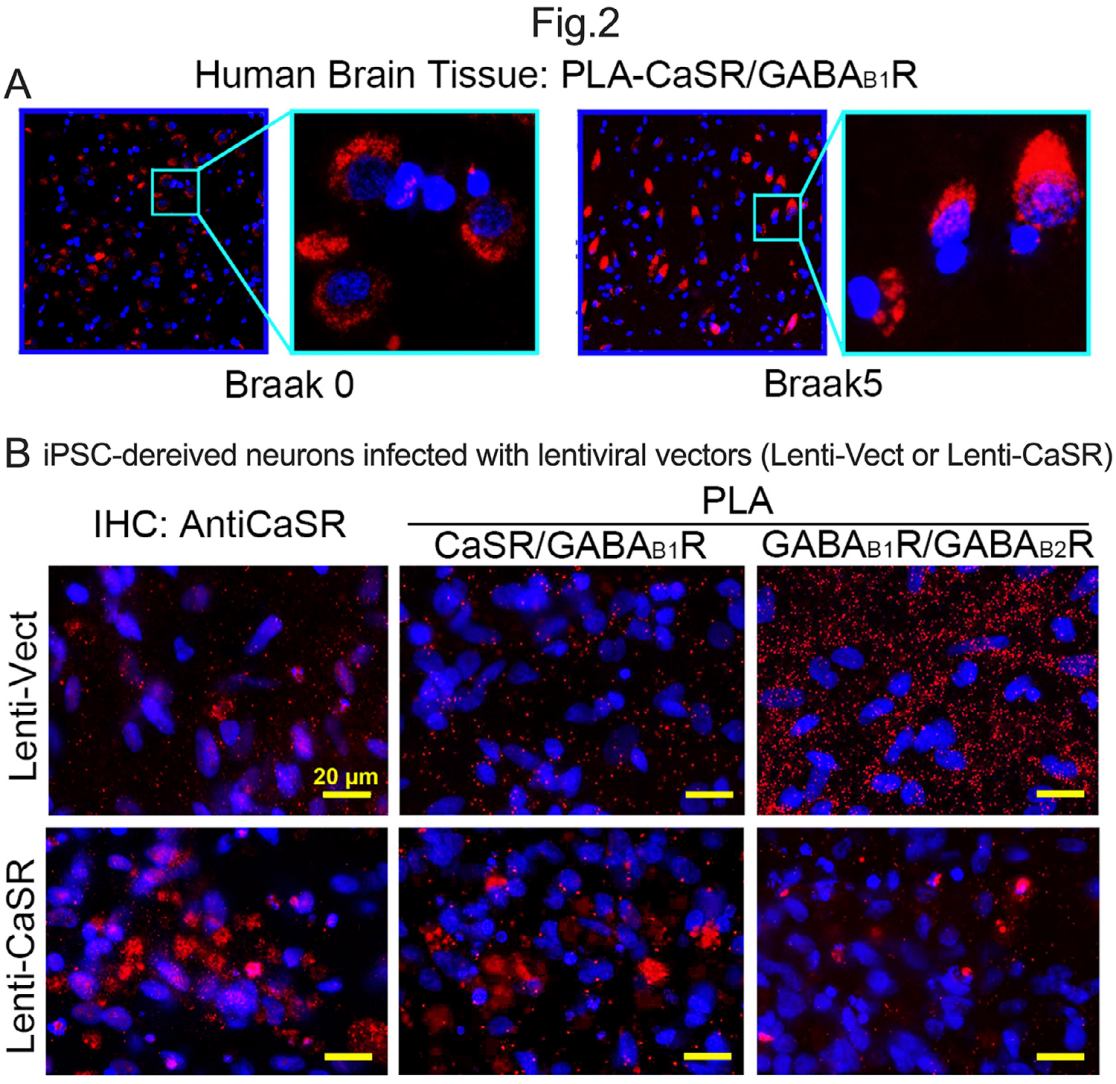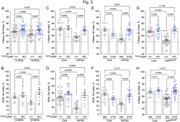# Neuronal Calcium‐Sensing Receptor Is A Druggable Target To Treat Alzheimer’s Disease

**DOI:** 10.1002/alz.088195

**Published:** 2025-01-03

**Authors:** Chia‐Ling Tu, Kota Kurisu, Zhiqiang Cheng, Stephanie R Miller, Atushi Mizuma, Jialing Liu, Jorge J Palop, Lea T. Grinberg, Midori A Yenari, Wenhan Chang

**Affiliations:** ^1^ San Francisco VA Medical Center, University of California San Francisco, San Francisco, CA USA; ^2^ Gladstone Institute of Neurological Disease, University of California San Francisco, San Francisco, CA USA; ^3^ Weill Institute for Neurosciences, University of California San Francisco, San Francisco, CA USA

## Abstract

**Background:**

Effective disease‐modifying regimens for Alzheimer’s Disease (AD) remain lacking due to insufficient understanding of its pathogenic drivers. It was shown previously that upregulation of the calcium‐sensing receptor (CaSR), an excitatory family C GPCR, induces neurodegeneration by interfering with the inhibitory γ‐aminobutyric acid (GABA) signaling following acute brain injuries (Ann_Clin_Transl_Neurol, 1:851‐66). Herein, we determined whether CaSR overexpression is causally associated with the AD.

**Method:**

(1) Proteomic profiles of hippocampal CA1 neurons in postmortem brains of subjects at progressive AD Braak stages (0, 1, 2, 4 and 6) were compared using a NanoString GeoMx profiler. (2) Proximity ligation assays (PLA) were performed to assess interactions of neuronal CaSR with type B GABA receptors (GABA_B1_R and GABA_B2_R). (3) Y‐maze and new object recognition (NOR) tests were performed to assess the impact of neuronal ablation of the *Casr* gene by Camk2a‐Cre‐mediated gene recombination (^Camk2a^CaSR^Δflox/Δflox^) or pharmacological inhibition of neuronal CaSR activities by daily subcutaneous injections of a brain‐permeable calcilytic (NPS‐2143, 20 µmole/kg) on cognitive functions of aging C57/B6 mice and two mouse models of early‐onset AD, 5XFAD (MMRRC_034840‐JAX) and hAPP^NL‐G‐F^ (Nature_Neuroscience, 5:661) mice.

**Result:**

The abundance of CaSR protein was significantly (p<0.0001) increased from the Braak stage 2 to 4/6 (Fig. 1A, C) and positively correlated (r^2^ = 0.2448 ‐ 0.4655, p<0.0001) with the abundance of APP, Aβ_1‐42_, BACE1, PSEN1, and phospho‐tau proteins (Fig. 1E‐I) in the somas of CA1 neurons (Fig. 1B) of the human brains. PLA showed increases in heterodimerization of CaSR with the GABA_B1_R in human brain from the Braak 0 to 5 (Fig. 2A) and in cultured hippocampal neurons overexpressing the CaSR by lentiviruses along with reduced GABA_B1_R/GABA_B2_R heterodimerization (Fig. 2B). While the control mice showed significant (p<0.001) cognitive declines from 12 to 18/20 months of age (MOA), these defects were prevented in the age‐matched ^Camk2a^CaSR^Δflox/Δflox^ or NPS‐2143‐injected mice (Fig. 3A, B). Similarly, the early cognitive declines in the 5XFAD (Fig. 3C‐F) and hAPP^NL‐G‐F^ (Fig. 3G, H) mice at 6 MOA were completely alleviated when the mice were bred into the ^Camk2a^CaSR^Δflox/Δflox^ background or injected with NPS‐2143.

**Conclusion:**

The neuronal CaSR is a critical driver of dementia by interfering with GABA_B_R signaling and a druggable target to prevent or treat AD.